# Comparison of non-magnetic and magnetic beads multiplex assay for assessment of *Plasmodium falciparum* antibodies

**DOI:** 10.7717/peerj.6120

**Published:** 2019-01-03

**Authors:** Bartholomew N. Ondigo, Gregory S. Park, Cyrus Ayieko, Donald D. Nyangahu, Ronald Wasswa, Chandy C. John

**Affiliations:** 1 Department of Biochemistry and Molecular Biology, Egerton University, Nakuru, Kenya; 2 Centre for Global Health Research, Kenya Medical Research Institute, Kisumu, Kenya; 3 Office of the Vice President for Research, University of Minnesota, Minneapolis, MN, USA; 4 Department of Zoology, Maseno University, Kisumu, Kenya; 5 Center for Global Infectious Disease Research, Seattle Children’s Research Institute, Seattle, WA, USA; 6 Department of Pediatrics, University of Washington, Seattle, WA, USA; 7 Global Health Uganda, Kampala, Uganda; 8 Ryan White Center for Pediatric Infectious Diseases and Global Health, Indiana University School of Medicine, Indianapolis, IN, USA

**Keywords:** Magnetic, Non-magnetic, Bio-Plex, Multiplex, Plasmodium falciparum antigens

## Abstract

**Background:**

New reagents have emerged allowing researchers to assess a growing number of vaccine-associated immune parameters. Multiplex immunoassay(s) are emerging as efficient high-throughput assays in malaria serology. Currently, commercial vendors market several bead reagents for cytometric bead assays (CBA) but relative performances are not well published. We have compared two types of bead-based multiplex assays to measure relative antibody levels to malarial antigens.

**Methods:**

Assays for the measurement of antibodies to five *Plasmodium falciparum* vaccine candidates using non-magnetic and magnetic fluorescent microspheres were compared for their performances with a Bio-Plex^200^ instrument. Mean fluorescence intensity (MFI) was determined from individuals from western Kenya and compared to known positive and negative control plasma samples.

**Results:**

*P. falciparum* recombinant antigens were successfully coupled to both non-magnetic and magnetic beads in multiplex assays. MFIs between the two bead types were comparable for all antigens tested. Bead recovery was superior with magnetic beads for all antigens. MFI values of stored non-magnetic coupled beads did not differ from freshly coupled beads, though they showed higher levels of bead aggregation.

**Discussion:**

Magnetic and non-magnetic beads performed similarly in *P. falciparum* antibody assays. Magnetic beads were more expensive, but had higher bead recovery, were more convenient to use, and provided rapid and easy protocol manipulation. Magnetic beads are a suitable alternative to non-magnetic beads in malarial antibody serology.

## Introduction

Antibodies to different *Plasmodium falciparum* antigens have been correlated in multiple immuno-epidemiological studies with protection from infection (Duarte et al., 2012; Dent et al., 2012) or disease (Lourembam & Baruah, 2012; Dodoo et al., 2011; Reiling et al., 2010). Cross-sectional studies of antibodies to one or two antigens have been used as markers to estimate past malaria exposure (Cook et al., 2012; Cook et al., 2010; Drakeley et al., 2005). However, such studies have often yielded conflicting results with some demonstrating a protective effect for antibodies to a specific antigen (Al-Yaman et al., 1996; Ogutu et al., 2009), while others do not show any association with malaria disease morbidity indicators (Murungi et al., 2013; Fowkes et al., 2010). Among the possible reasons for the inconsistent findings are differences in standardization and analytical approaches (Fowkes et al., 2010).

In many aspects, multiplex fluorescent microsphere assays are a more efficient method to detect plasma antibodies to *P. falciparum in* comparison to a traditional ELISA (for example, sample requirements and handling). The capacity to measure relative levels of antibodies to multiple antigens could ultimately increase uniformity for comparative reasons. On this front, there has been a surge in the development of multiplex methodologies as reliable replacements of ELISAs (Cham et al., 2008; Lal et al., 2005; Verkaik et al., 2008). These assays utilize microsphere beads, which are conjugated to specific capture reagents, including conjugation-ready bead sets carrying either avidin, carboxylate groups (COOH) or oligonucleotide adapters (Reslova et al., 2017; Elshal & McCoy, 2006). Recently, magnetic microspheres (beads) have become available as an alternative option to non-magnetic beads. Magnetic beads are made of a core of magnetic material consisting of iron oxide and an internal array of dyes which color code the beads, thus allowing multiplexing (Verkaik et al., 2008; Dunbar & Li, 2010; Houser, 2012). Additional similarities and differences between the two bead types are as shown in Table 1.

The introduction of magnetic beads demands validation with a Bio-Plex^100∕200^ instrument that uses laser excitation and digital signal processing technology. No malaria specific antibody studies have directly compared multiplex assay results obtained from non-magnetic with the newer, magnetic beads. The purpose of this study was to determine if antibody quantification assays using magnetic beads provided similar results to assays using non-magnetic beads on the Bio-Plex^200^ platform. Additionally, we explored the effect of long-term storage on MFI values obtained from non-magnetic bead multiplex assays. To address these objectives, a 5-plex and 9-plex assay were respectively employed to measure antibodies to *P. falciparum* vaccine candidate antigens from the plasma of individuals from western Kenya and North America never exposed to malaria (negative control samples).

## Materials and Methods

### Non-magnetic and magnetic beads

The carboxylated SeroMAP™ microspheres (non-magnetic) product numbers: S005, S015, S025, S030, S040, S045, S050, S055, and S060; and carboxylated MagPlex™ microspheres (magnetic) beads, product numbers: MC10029-01, MC10036-01, MC10045-01, MC10054-01 and MC10065-01 were used. Bead stock suspensions were at 1. 25 ×10^7^ beads mL^−1^ and were stored at 2–4 °C. Both beads and sheath fluid were obtained from Luminex (Austin, Texas, USA).

**Table 1:** Similarities and differences between non-magnetic and magnetic beads used in multiplex assays. The left column indicates the characteristic being compared across the two types of beads.

Characteristic	Non-magnetic beads	Magnetic beads
Provision for a scalable coupling protocol	Yes	Yes
Size of bead from xMAP technology	5.6 µm spherical beads	6.5 µm spherical beads
Composition and property	Polystyrene beads, show a non-magnetic behavior	Super paramagnetic particles embedded in a non-magnetic polymer matrix, show a non-magnetic behavior, unless when exposed to a magnetizing field.
Ease of processing	Centrifugation and vacuum workflow utilized	Requires magnetic separator
Washing	Requires filter plates and vacuum filtration	Requires an automated Bio-Plex wash station and/or a simple hand held magnetic plate can also be used
Type of assay supported	Commercially available and laboratory developed assays	Commercially available and laboratory developed assays

### Instrumentation

The antibody measurements were performed using a Bio-Plex^200^ (Bio-Rad, Hercules, CA, USA). The Bio-Plex^200^ consists of a Luminex 200 suspension array reader, high-throughput fluidics system, a microplate platform, monitor and computer operated with BPM 5.1 software (Bio-Rad). All washing steps of magnetic beads were carried out on a HydroFlex™ microplate washer (Tecan, Männedorf, Switzerland) using a magnetic plate carrier, while washing of non-magnetic beads was performed using a millipore vacuum manifold. Plates were incubated on an IKA micro-titer shaker ^®^MTS (Wilmington, NC, USA). During the coupling procedures, the magnetic microspheres were captured using a DynaMag magnetic separator (Life Technologies AS Oslo, UK). All centrifugation steps were carried out in an Eppendorf centrifuge.

### Plasma samples

For all multiplex bead assays, a plasma pool made of 30 plasma samples from adults living in a Ugandan area of seasonal malaria transmission (positive control pool samples Idro et al., 2005) while seven plasma samples from malaria-naive North American individuals were used as negative controls. To assess the utility of the coupled beads, a broad range of plasma test samples from 32 individuals from a malaria endemic area of western Kenya (John et al., 2005) were also used. Comparisons of both coupled bead types were performed using the same plasma samples. To compare the instrument detection limit (IDL) between the bead types 8 duplicate serially diluted positive control plasma at the following dilutions: 1: 50, 1:100, 1:200, 1:400, 1:1,000, 1:2,000, and 1:4,000 were analyzed by a 5-plex assay—(AMA-1, EBA-175, MSP-1(19), MSP- 1(42) and MSP-3). In addition, comparison of freshly coupled and 18-months stored coupled beads was performed by use of Bio-Plex 200. Written informed consent was obtained from the study participants or, in the case of minors, from their parent or guardian. Ethical approval was obtained from Kenya Medical Research Institute National Ethical Review Committee (protocol number 2101) and the Institutional Review Board of University of Minnesota.

### *P. falciparum* recombinant and peptide antigens

The recombinant *P. falciparum* antigens used for testing were apical membrane antigen-1 (AMA-1, full length ectodomain FVO strain); erythrocyte-binding antigen (EBA-175, non-glycosylated region II); glutamate rich protein (GLURP, conserved non-repeat N-terminal region, amino acids 25–514, R0; and repeat C-terminal region, amino acids 705–1178, R2, 3D7 strain); liver stage antigen (LSA-NRC, C-terminal region, amino acids 1628 to 1909, 3D7 strain); merozoite surface protein-1 (MSP-1_19_, E-KNG variant; MSP-1_42_ FVO strain); merozoite surface protein-3 (MSP-3, C-terminus, FVO strain) and recombinant thrombospondin-related adhesion protein (TRAP, 3D7 strain). Recombinant AMA-1, LSA-NRC and TRAP were expressed in *Escherichia coli* and provided by David E. Lanar, Walter Reed Army Institute for Research. Recombinant MSP-1_42_ and MSP-3 were expressed in *E. coli*, and EBA-175 was expressed in *Pichia pastoris*, and provided by David Narum, National Institutes of Health. Recombinant GLURP-R0 and GLURP-R2 were expressed in *E. coli* and provided by Michael Theisen, Statens Seruminstitut, Copenhagen, Denmark. Recombinant MSP-1_19_ expressed in *Saccharomyces cerevisiae*, was provided by the Malaria Research and Reference Reagent Resource Center (Manassas, VA), and originally deposited there by David Kaslow.

### Coupling of recombinant antigens to non-magnetic and magnetic microspheres

Prior to use, all buffers were brought to room temperature, EDC and Sulfo-NHS were desiccated at room temperature for approximately 1 h, and both types of microspheres were vortexed for 30 s then sonicated for 15 s *. P. falciparum* antigens were directly coupled to non-magnetic beads and each to a unique bead set, using a previously described procedure (Ondigo et al., 2012). We determined the optimal amounts of antigen by coupling 612,500 beads to differing amounts (0.5, 1, 2, 5 and 10 µg) of each antigen and the optimal antigen amount was the amount that yielded the highest average MFI value. The optimal amount of antigen coupled to beads were: GLURP-R0 and GLURP-R2 was 0.5 µg, MSP- 1_42_ FVO and MSP-3 FVO was 1 µg; AMA-1 FVO and EBA-175 was 2 µg; and for MSP-1_19_ EKNG, LSA-NRC and TRAP were at 10 µg for each of the 612,500 beads. Magnetic microspheres were coupled to the same amount of *P. falciparum* antigens as non-magnetic microspheres, as per the manufacturer’s protocol. Magnetic beads were pelleted using a magnetic separator.

### IgG Cytometric bead assay to *P. falciparum* antigens coupled to non-magnetic beads

All reagents and samples were brought to room temperature before use for both types of beads and plasma samples were diluted before use. Non-magnetic beads were diluted with PBNT (0.1% BSA, 0.05% Tween 20, 0.05% sodium azide in PBS) and used at a working concentration of 1,000 beads/region/well. Bead stocks were then combined in a 15 ml amber conical tube. 96 well-millipore microtiter plates (MABVN 1250, Millipore corporation, Billerica, MA) were pre-wetted with 100 µl of PBNT/well and aspirated using a millipore vacuum manifold and 50 µl of working bead solution was transferred to each well. Plasma samples were thawed at room temperature, mixed and centrifuged at 16,000 g for 3 min then diluted to 1:100 in buffer (1xPBS, 1% BSA, 0.05% Tween 20, 0.05% sodium azide, 0.5% polyvinyl alcohol, and 0.8% polyvinyl pyrrolidone). Fifty µl of diluted plasma was added into each well of a microtiter plate in duplicate. The plasma was mixed with the beads three times by pipetting up and down and the plates incubated in the dark on a shaking micro- plate shaker (IKA^®^ MTS, Wilmington, NC, USA) at 600 rpm for 30 s, followed by 300 rpm for 30 min at room temperature (RT). Plates were aspirated using a millipore vacuum manifold, washed twice with 100 µl/well of PBNT, and beads resuspended in 50 µl PBNT. Fifty µl of goat anti-human IgG (1:1,000 in PBNT, F (ab‘)2 fragment-R-phycoerythrin (Sigma, P-8047 #SLBH5768V St. Louis, MO) was added to each well, and incubated in the dark with shaking at 600 rpm for 30 s, followed by 300 rpm for 30 min. Plates were aspirated using a millipore vacuum manifold and washed twice with 100 µl/well PBNT. The beads were resuspended in 100 µl PBNT and finally analyzed on a Bio-Plex^200^ (Bio-Rad, Hercules, CA, USA) equipped with Bio-Plex Manager 5.0 software (Bio-Rad). Samples were analyzed with the following Bio-Plex^200^ settings: 100 beads per region, timeout time was set at none, 50 µL sample volume, and DD gate 5,000–2,5000, default settings and the RP1 target were turned on.

### IgG Cytometric bead assay to *P. falciparum* antigens coupled to magnetic beads

The magnetic-based assays were carried out according to the protocol as provided by the manufacturer. Briefly, 50 µl of working bead solution (1,000 beads/region/well) was transferred to each Greiner 96-well microtiter black, flat bottom plate (Greiner Bio-One, Frickenhausen, Germany). The beads were incubated in the dark on a microplate shaker at 600 rpm for 30 s, followed by 300 rpm for 30 min with 1: 100 diluted plasma samples added in duplicate at room temperature. Wells were washed twice with an automated HydroFlex™ microplate washer (Tecan, Männedorf, Switzerland) using 100 µl of PBNT, and beads resuspended in 50 µl PBNT. Fifty µl goat anti-human IgG (1:1,000 in PBNT, F (ab)2 fragment-R-phycoerythrin was added to each well. Wells were washed twice with 100 µl PBNT with an automated microplate washer and beads resuspended in 100 µl PBNT and finally analyzed on a Bio-Plex^200^ (Bio-Rad, Hercules, CA, USA) equipped with Bio-Plex Manager 5.0 software (Bio-Rad) for median fluorescence intensity (MFI). Analyses were performed with a minimum count of 100 beads per region as per the manufacturer’s instructions.

### Statistical analysis

After subtraction of the background blank, the mean of results from the duplicate wells was reported as the median fluorescence intensity minus background (MFI–BG). To obtain the cut off value for positivity, three standard deviations (SD) were added to the average MFI of the negative controls. Mann Whitney test was used to compare between MFI values from non-magnetic and magnetic beads. In addition, Spearman correlation coefficients (Rho) was used to compare MFI values obtained from stored coupled beads for 18 months and freshly coupled beads. A *P*-value of <0.05 was considered to be statistically significant. Statistical analysis was performed using GraphPad Prism version 5.0 (GraphPad Software Inc., San Diego, CA, USA).

## Results

### Comparison of MFI values of non-magnetic and magnetic beads by Bio-Plex^200^ instrument

To assess the performance of the multiplex assays the range of fluorescence generated was used as an indicator of the effectiveness of antigen coupling to the beads. The mean MFIs (SD) ranged from low values (for blanks) to high values (for positive plasma controls) for both non-magnetic and magnetic beads. These ranges of values suggested successful coupling reactions for each of the five *P. falciparum* recombinant antigens to the two bead types (Table 2). A comparison of the fluorescence generated with the negative controls in each of the bead types showed that there was no statistical difference between non-magnetic and magnetic beads, for each of the analyte measured (all *P* > 0.05) (Table 2).

**Table 2:** MFI values from non-magnetic and magnetic beads in a 5-plex Bio-Plex assay. MFI values (SD), generated by non-magnetic and magnetic beads for blanks, negative controls (*n* = 7) and positive controls (pooled plasma samples) in a 5-plex assay (SD –Standard Deviation).

Analyte	AMA-1		EBA-175		MSP-1_19_		MSP-1_42_		MSP-3	
	Nonmagnetic beads	Magnetic beads	Nonmagnetic beads	Magnetic beads	Nonmagnetic beads	Magnetic beads	Nonmagnetic beads	Magnetic beads	Nonmagnetic beads	Magnetic beads
Blanks (SD)	3.0 (1.4)	5.5 (0.7)	1.0 (0)	5.0 (0)	0.5 (0.7)	5.0 (0)	1.0 (0)	3.5 (0.7)	0.5 (0.7)	4.0 (0)
Negative controls (SD)	13.0 (9.5)	47.5 (60.6)	27.0 (13.6)	48.8 (22.7)	3.9 (2.9)	13.3 (5.5)	15.0 (10.9)	13.5 (7.5)	36.4 (20.8)	25.6 (19.1)
Positive controls (SD)	7,706.0 (503.5)	5,463.8 (846.1)	6,774.8 (446.5)	4,759.0 (304.8)	7,376.8 (382.2)	4,210.0 (42.4)	8,747.5 (286.4)	4,499.0 (257.4)	385.8 (3.2)	533.3 (48.4)

### Instrument Detection Limit of non-magnetic and magnetic beads by Bio-Plex^200^ instrument

**Figure 1:**
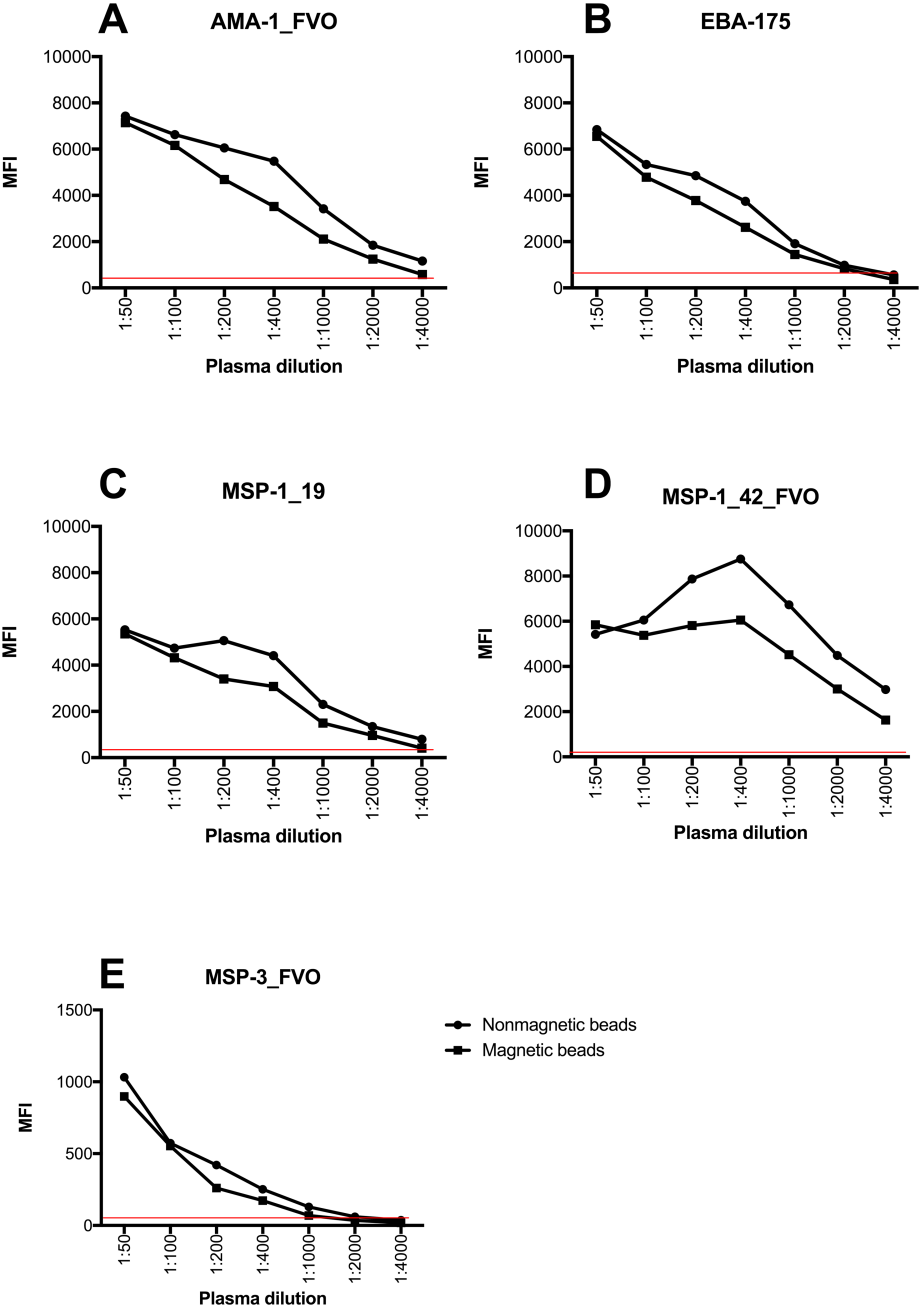
Instrument detection limit over a series of plasma dilutions on a 5-plex assay with non-magnetic and magnetic beads. The red line indicates cut off for positivity that was determined using average MFI plus 3 standard deviations of negative controls. *Y*-axis indicates the linear MFI values while *x*- axis is a range of plasma dilutions tested (1: 50 –1:4,000). MFI values of *Y*-axis for (A–D) range from 0–10,000 while for (E) range from 0–1,500.

The instrument detection limit (IDL) was defined as the analyte concentration that would be required to produce a signal greater than three times the standard deviation of the noise level (negative controls) by both non-magnetic and magnetic beads. IDL provided the lowest quantity of an analyte that could be distinguished above the negative control samples. This experiment provided a proxy measure of accuracy of the two sets of beads from the raw MFI values generated. Both non-magnetic and magnetic beads exhibited a wide dynamic range and had the ability to read concentrations down to the minimum reported concentration for each of the analytes. Most importantly, the two bead types provided similar detection limits at low plasma concentrations for all analytes (Fig. 1, Supplemental Information 1).

### Comparison of MFI values between non-magnetic and magnetic beads on a 5-plex assay

**Figure 2:**
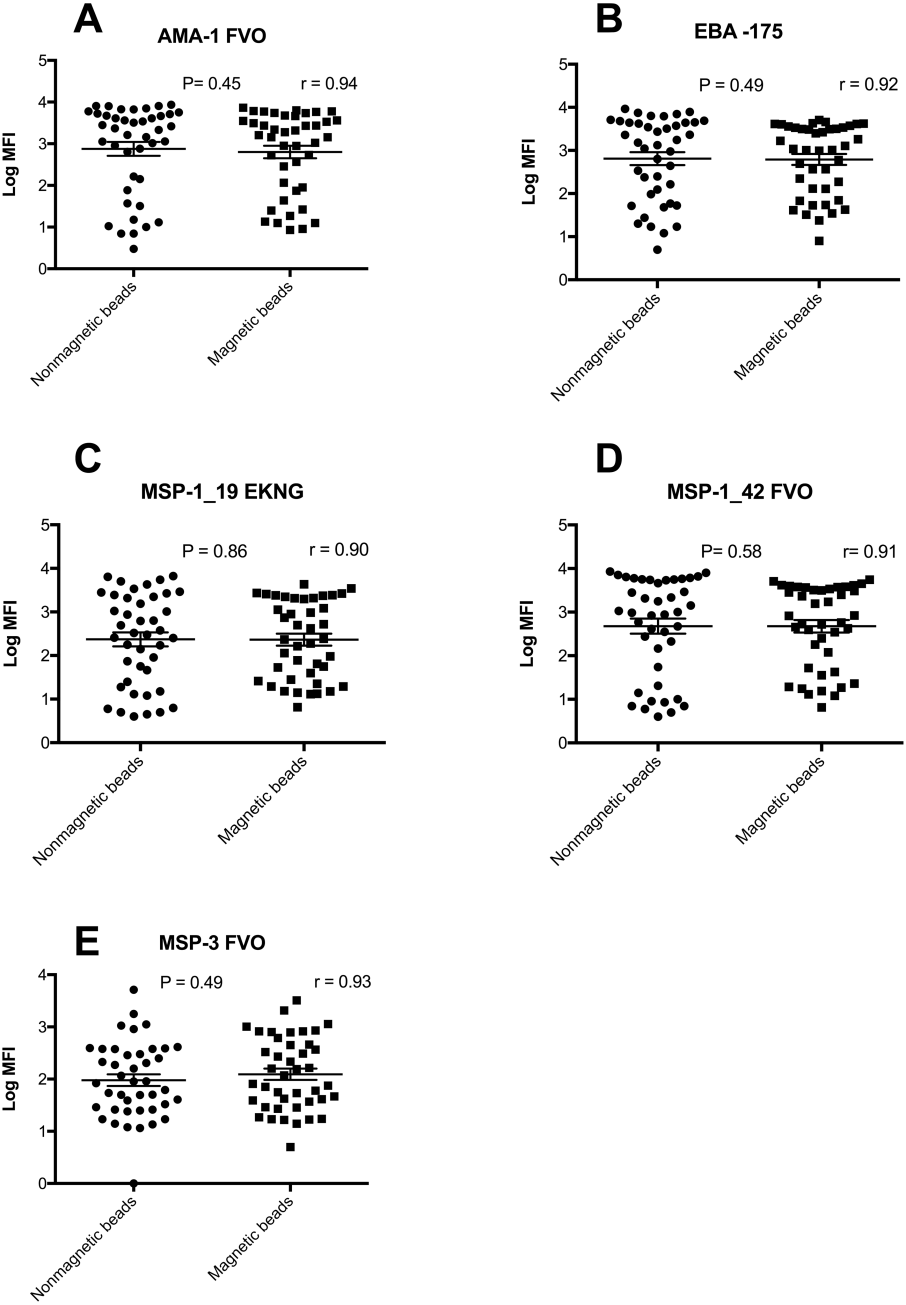
Log MFI values of non-magnetic and magnetic beads in a 5-plex assay. Log MFI values obtained from individuals plasma samples generated using non-magnetic and magnetic beads in a 5-plex assay with horizontal lines indicating the median values and the 25th and 75th percentiles (*n* = 42). The *Y*- axis has logarithmic values of MFI, while *x*-axis indicate the type of beads.

A comparison of ranges of MFI values obtained in a range of samples from individuals from western Kenya and North Americans showed that the two bead types had equivalent MFI values (Fig. 2, Supplemental Information 1) (all *P* > 0.05). In addition the number of positive responders for AMA-1, EBA-175, MSP-1_19_, MSP-1_42_ and MSP-3 IgG antibodies were almost identical (data not shown).

### Comparison of post coupling microsphere recovery for non-magnetic and magnetic beads

To quantify bead recovery obtained we counted the number of microspheres recovered after the coupling reaction using a cell counter and a hemocytometer and compared this count with the starting corresponding number of beads multiplied by 100 percent. Bead recovery ranged from 63–76% (AMA-1–70%, EBA-175–64%, MSP-1(19)–76%, MSP-1(42)–63% and MSP-3–66%) for non- magnetic beads. Bead recovery in magnetic beads, ranged from 83–92% (AMA-1–83%, EBA-175–84%, MSP-1 (19)–86%, MSP-1 (42)–89% and MSP-3–92%). Our findings indicate that magnetic beads had higher yields microsphere recoveries post-coupling for each of the five coupled *P. falciparum* antigens. On the contrary, a lower non-magnetic bead recovery suggested a higher loss of non-magnetic beads during the coupling process, which ultimately limits the amount available for multiplex serological assays.

### Stability of antigen coupled non-magnetic beads with a Bio-Plex^200^ instrument

**Figure 3:**
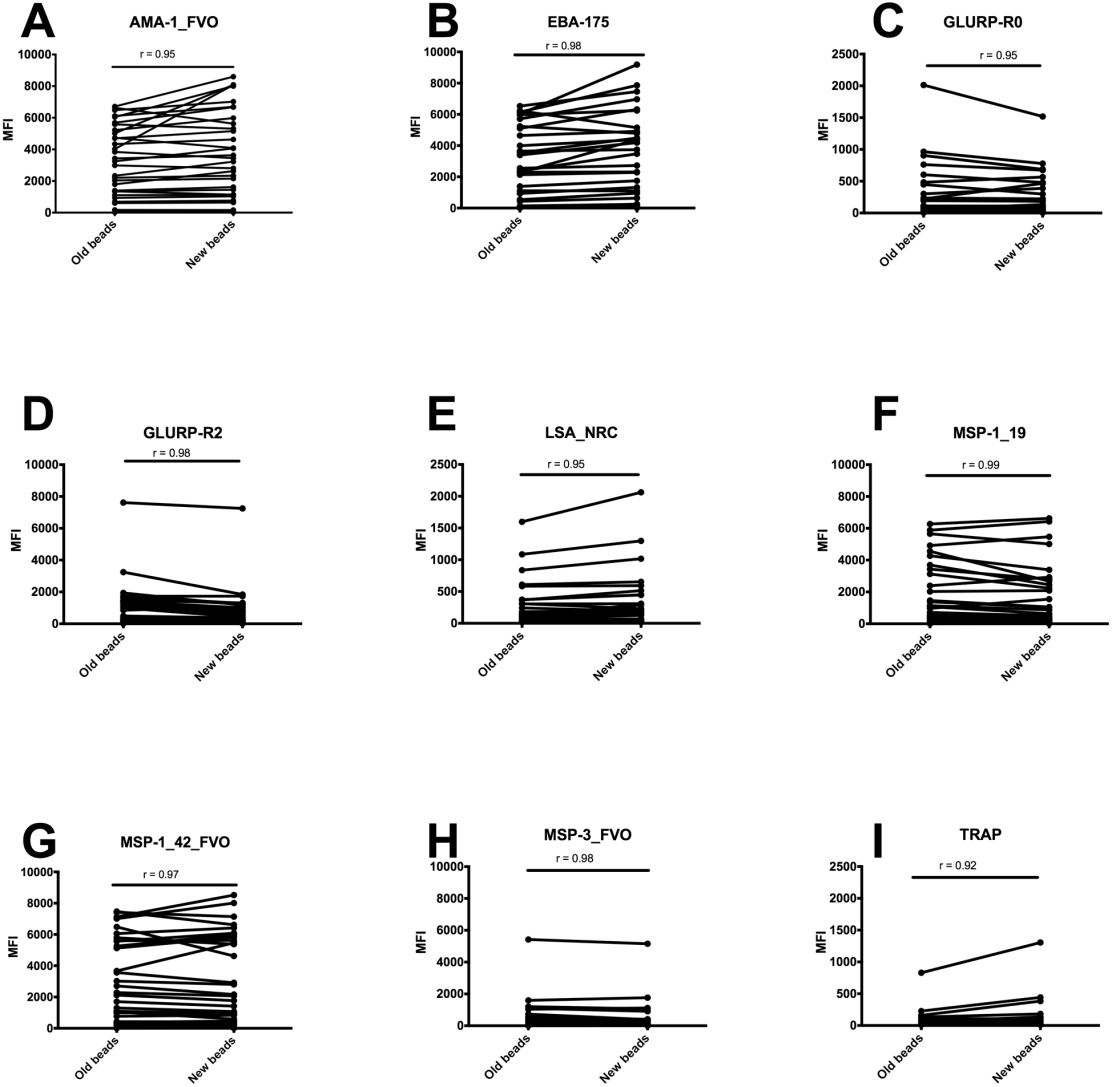
MFI values of freshly coupled non-magnetic beads and after 18 months of storage at 4° C in a 9-plex Bio-Plex assay. MFI values of freshly coupled non-magnetic beads and after 18 months of storage at 4 °C in a 9-plex assay obtained from individual plasma samples in western Kenya, horizontal lines indicating the change in MFI value between the two sets of beads (*n* = 32). The *Y* axis scale has a linear MFI values ranging from 0–10,000 for (A, B, D, F, G, H) and 0–2, 500 for (C) (E) and (I). *X*-axis indicate the status of non-magnetic beads.

To determine if antigens decoupled from the beads during 18 months storage at 4 °C, antibodies to nine *P.  falciparum* antigens were measured in plasma samples from individuals from malaria endemic area of western Kenya, using freshly coupled beads and beads coupled 18 months ago and stored at 4 °C during this time. Percentage bead aggregation between the types was higher in beads that had been stored for 18 months (data not shown). Freshly coupled beads had similar MFI values to beads that had been stored for 18 months for each of the antibody measured, and all antibody measurements were highly correlated with Spearman correlation rho values, r for all >0.9 (AMA-1, 0.95; EBA-175, 0.98; GLURP-R0, 0.95; GLURP-R2, 0.98; LSA-NRC, 0.95; MSP-1_19,_ 0.99; MSP-1_42,_ 0.97; MSP-3, 0.98; and TRAP, 0.92, (all *P* > 0.05) Fig. 3, Supplemental Information 1). We did not measure stability in magnetic beads as these were new to our experiments, and we did not have storage time to assess longevity of responses. Future experiments are planned to assess longevity of antigen coupled magnetic beads.

## Discussion

This paper shows that non-magnetic and magnetic beads perform similarly when used to measure antibodies to *P. falciparum* antigens. Since the two bead assays are comparable in this respect, other parameters such as ease of use, quick magnetic bead separation and availability should determine suitability of the assay. For both non-magnetic and magnetic beads antigen coupling was successful, the non-specific background was low for blank samples and, the specific signal intensity was high for positive control samples. Also, they were able to discriminate different concentrations of antibodies on serially diluted test samples. Above all, the antigen coupled magnetic microspheres had higher efficiency of bead recoveries. The findings suggest that magnetic bead assays would provide a powerful tool for detection and quantification of *P. falciparum* antibodies.

The results show that both types of beads were comparable (Fig. 2). MFI values were higher with non-magnetic beads compared to magnetic beads for all the analytes except MSP-3 when positive control samples were analyzed (Table 2). Although a higher MFI does not necessarily reflect a better or more valid finding, it may provide a broader dynamic range (Verkaik et al., 2008). Magnetic beads are advantageous for malaria serology due to their ability to be manipulated easily during couplings and with the development of automatic washing system; the beads could be trapped on the bottom of the plate (Table 1). A critical step of bead-based assays is bead recovery. Improvement in percent bead recovery, maximizes the yield, results in a larger amount of reagents available to be used when performing the assays and reduction on the analysis time for each plate. Multiple centrifugation results in significant bead loss, which ultimately increases the cost of performing the assay. We observed a 13–26% greater bead recovery with magnetic compared to non-magnetic beads. However, magnetic beads are more expensive compared to non-magnetic beads (cost for a vial of 1.25 × 10^7^ beads/ml, $726.00 vs. $438.00 (Bio-Rad Corporation) as at the time of this manuscript submission). In addition, coupling magnetic beads to proteins of interest does not require centrifugation and sedimentation processes. Elimination of centrifugation increases bead recoveries and ease of the assay, making it simple and convenient to the user. Further, use of magnetic beads may overcome limitations associated with non-magnetic beads such as leaking, clogging of micro-plates after washing steps and the need for expensive filter plates. Further, development of automated plate washers for use with magnetic beads further facilitates rapid and accurate testing and overcomes limitations in effectively washing samples using non-magnetic beads. In summary, the process of coupling magnetic beads to proteins of interest is more straightforward and efficient, reduces bead loss, and does not require the use of disposable filter plates. Although magnetic beads are more expensive than non-magnetic beads, the price is comparable when considering the efficiency and use of standard microtiter plates.

In a prior study, storage of coupled non-magnetic beads for seven months at 4 °C in the dark resulted in a decrease in MFI, and it was recommended that lyophilization (Cham et al., 2008) could be a suitable alternative to minimize disassociation of the antigen(s) from the beads. In this study, coupled non-magnetic beads were stored at 4 °C for 18 months with no significant loss of sensitivity for antibodies to *P. falciparum* (Fig. 3), suggesting that at least for these antigens, engagement between the beads and proteins lasts for prolonged periods at 4 °C, though bead aggregation is increased.

Overall, the magnetic based Bio-Plex assays offer a novel multiplexed approach to assess *P. falciparum* antibody patterns in humans plasma samples, which will help advance our understanding of malaria biology and associated host immunological responses. However, this study had a limited sample size and a few analytes were tested because of design aspects based on the amount of stored reagents available for use. Future work should assess the effect of storage of coupled magnetic beads on antibody detection.

## Conclusions

The magnetic beads performed similarly in *P. falciparum* antibody assays and provide a suitable alternative to non-magnetic beads for measurement of antibodies to *P. falciparum* antigens. In addition, use of magnetic beads provide the capacity to evaluate a higher number of *P. falciparum* analytes due to improved bead recoveries.

##  Supplemental Information

10.7717/peerj.6120/supp-1 Supplemental Information 1 Beads raw data

## Additional Information and Declarations

### Data Availability

The following information was supplied regarding data availability: The raw data are available in the Supplemental File.
